# Whole Organ Tissue Vascularization: Engineering the Tree to Develop the Fruits

**DOI:** 10.3389/fbioe.2018.00056

**Published:** 2018-05-14

**Authors:** Alessandro F. Pellegata, Alfonso M. Tedeschi, Paolo De Coppi

**Affiliations:** ^1^Stem Cells and Regenerative Medicine, Great Ormond Street Institute of Child Health, University College London, London, United Kingdom; ^2^SNAPS, Great Ormond Street Hospital for Children NHS Foundation Trust, University College London, London, United Kingdom

**Keywords:** organ, tissue engineering, vascularization, stem cells, regenerative medicine, decellularization, angiogenesis, endothelial cells

## Abstract

Tissue engineering aims to regenerate and recapitulate a tissue or organ that has lost its function. So far successful clinical translation has been limited to hollow organs in which rudimental vascularization can be achieved by inserting the graft into flaps of the omentum or muscle fascia. This technique used to stimulate vascularization of the graft takes advantage of angiogenesis from existing vascular networks. Vascularization of the engineered graft is a fundamental requirement in the process of engineering more complex organs, as it is crucial for the efficient delivery of nutrients and oxygen following *in-vivo* implantation. To achieve vascularization of the organ many different techniques have been investigated and exploited. The most promising results have been obtained by seeding endothelial cells directly into decellularized scaffolds, taking advantage of the channels remaining from the pre-existing vascular network. Currently, the main hurdle we need to overcome is achieving a fully functional vascular endothelium, stable over a long time period of time, which is engineered using a cell source that is clinically suitable and can generate, *in vitro*, a yield of cells suitable for the engineering of human sized organs. This review will give an overview of the approaches that have recently been investigated to address the issue of vascularization in the field of tissue engineering of whole organs, and will highlight the current caveats and hurdles that should be addressed in the future.

## Introduction

The availability of whole organs for transplantation still represents a significant burden, while the clinical demand continues to increase. Indeed, the number of patients that are eligible for transplant therapy is likely to grow further in the future. This is thanks to the continued development of medical technologies that can preserve life, allowing patients to live with chronic conditions which were previously fatal. Various congenital and acquired pathologies result in organ failure, for which the only cure is organ transplantation. Donor organ shortages, and complications associated with life-long immunosuppression related to allogeneic transplantation, result in significant morbidity and mortality (Orlando et al., 2012).

**Figure 1:**
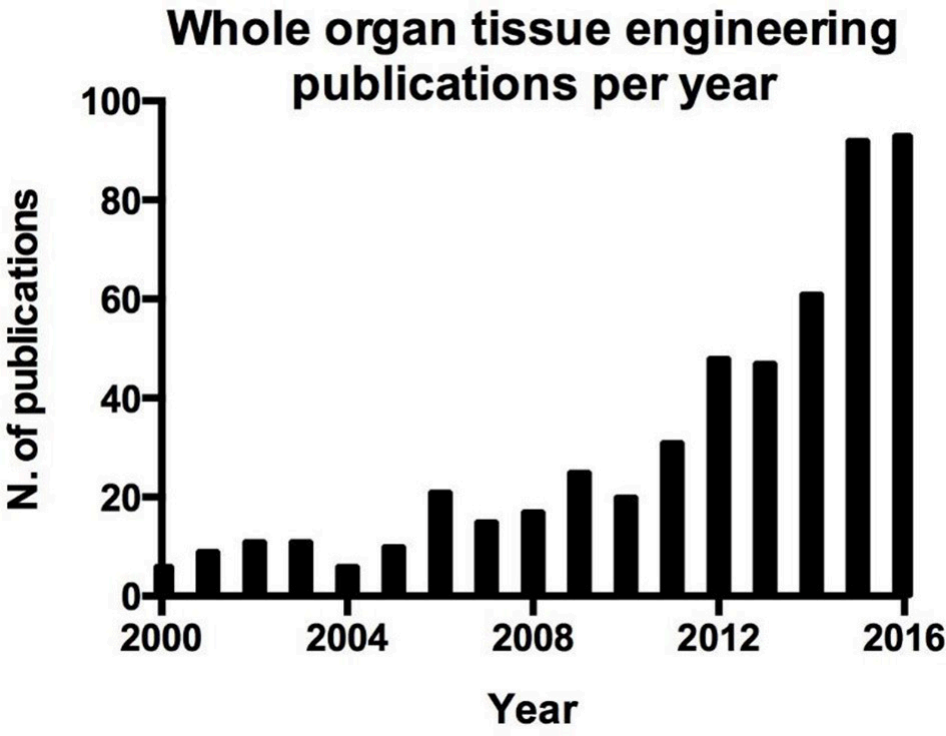
Number of publication per year on whole organ tissue engineering resulting from a search on Pubmed.

Regenerative medicine, in particular tissue engineering aims to regenerate a tissue or organ that has lost its function. This field of research takes advantage of cells, scaffolds and stimuli, delivered through a bioreactor to the growing organ *in vitro* (Tresoldi et al., 2015). Tissue engineering as an approach could represent the best route available to overcome the hurdles related to organ transplantation. Over the last years, interest in this topic has grown, as demonstrated by the numerous studies addressing tissue engineering of whole organs (Figure 1). To restore the function of an organ it is vital that all compartments are engineered (Badylak et al., 2011), since the overall function of an organ is due to the synergy of its individual compartments e.g., epithelia, mesoderm, parenchyma and vasculature. It can be argued that the vasculature, in particular, is of great importance in whole organ engineering, and represents the major point of communication between the organ and the rest of the body. For example in organs that exert an endocrine function chemicals are released into the blood stream, while more importantly, the vasculature delivers oxygen and nutrients to the organ, essential for survival. This latter aspect is fundamental in the process of whole organ tissue engineering since the delivery of oxygen in an avascular tissue would be limited to a few hundreds μm by gas diffusion (Jain et al., 2005). This would certainly result in necrosis which would hamper the *in vitro* growth of organs and limit survival post-transplantation. Ideally, the vasculature of the tissue engineered organ should be directly connected to the host vasculature, optimally this would take place at the time of organ grafting by direct anastomosis. Alternatively, the graft could be subjected to an environment that promotes angiogenesis, if rapid ingrowth of host vasculature could be stimulated, over a period short enough to avoid tissue necrosis of the graft, this may provide a vascular network capable of sustaining graft survival.

**Figure 2:**
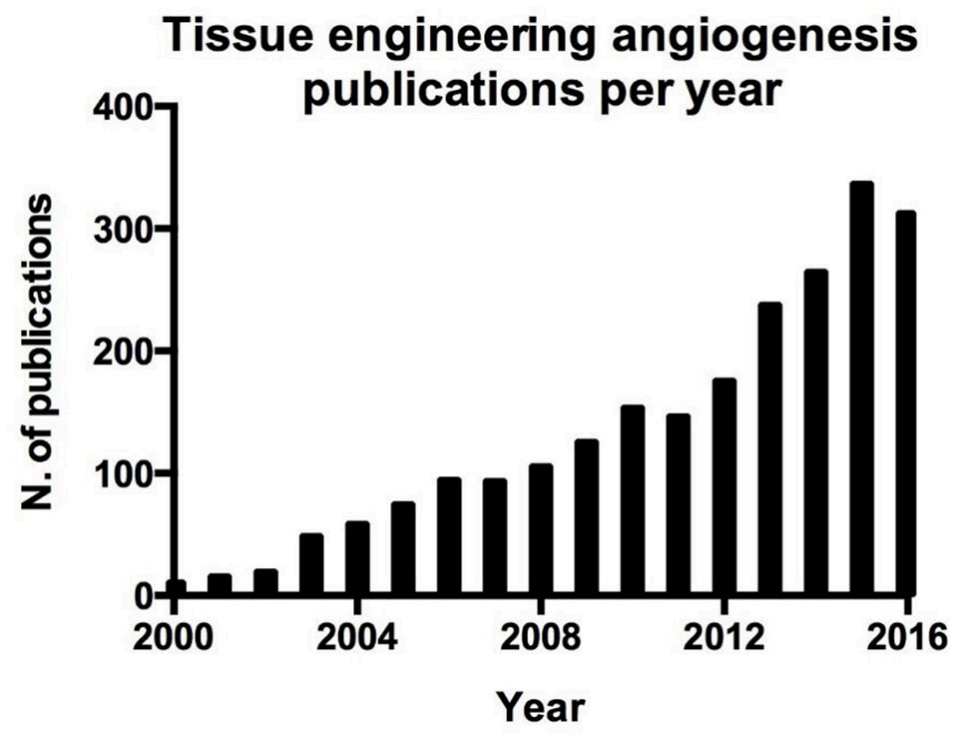
Number of publication per year on angiogenesis in tissue engineering resulting from a search on Pubmed.

Blood vessel function is not only limited to the above mentioned functions, indeed endothelial cells play an active role in orchestrating the processes involved in tissue repair (Ding et al., 2011; Takebe et al., 2013; Hu et al., 2014; Pellegata et al., 2015; Poulos et al., 2015; Ramasamy et al., 2015). This aspect is crucial in the regeneration and engraftment processes of whole organ engineering and can be easily demonstrated by the parallel interest in whole organ tissue engineering (Figure 1) and angiogenesis in tissue engineering (Figure 2).

In order to engineer whole organs that can function and survive upon grafting, it is essential to incorporate a functional endothelium. Establishing a properly organized vascular network that features vessels of the correct size, protruding evenly throughout the whole organ will have a huge impact on translation of tissue engineered organs into clinical practice. The optimal scenario would be for researchers to establish techniques for the development of endothelial layers, thus providing a barrier with vasomotility and a site for perfusion which matches the specific typology of the target organ in terms of endothelial pattern, such as normal, fenestrated or sinusoidal (Rafii et al., 2016).

Although organ vascularization represents a significant bottleneck to clinical translation, many different and promising approaches have been investigated. This review will provide an overview of the different strategies that have been employed, analyzing the state of the art techniques applied to the major organs of the body.

## Whole organs decellularization

Decellularization is the complete removal of all cellular and nuclear material from a tissue while preserving its extracellular matrix (Gilpin and Yang, 2017). The process is usually achieved by means of detergents and enzymes, coupled with physical stress. Nearly every tissue of the human body has been decellularized, and very recently, whole human limbs have been used to produce acellular scaffolds (Gerli et al., 2018). This technique has the unique advantage of generating a scaffold that closely resembles the native environment from both a biochemical and anatomical point of view (Crapo et al., 2011). Acellular matrices allow cellular growth and functional differentiation without triggering an immune response, even in the case of xenogeneic transplantation (Fishman et al., 2013).

The natural and obvious evolution of this technique has been the decellularization of whole organs (Scarritt et al., 2015). This approach represents the easiest way to obtain a scaffold which exactly mimics the complex structure of an organ, an aspect that is crucial to rebuild the organ and restore its function. Moreover, organs that are deemed medically unsuitable for transplantation due to poor condition, could represent a potential source of organs for the generation of decellularized scaffolds for tissue engineering (Peloso et al., 2015; Verstegen et al., 2017).

Interestingly, it has been shown that among the different compartments preserved within decellularized organs, the extracellular matrix of the vasculature is unaltered and retains its structure, resulting an easy route to deliver cells evenly throughout the whole organ. Preservation of the vasculature has been demonstrated in different organs such as intestine (Totonelli et al., 2012), lung (Maghsoudlou et al., 2013), liver (Maghsoudlou et al., 2016), heart (Ott et al., 2008), and kidney (Song et al., 2013; Bonandrini et al., 2014).

## Cells for whole organ tissue engineering

The microvasculature is an essential feature of the organ, needed to transport nutrients, blood cells, oxygen and waste products. This network of complex vessels is formed by a process of angiogenesis and neovascularization. It is composed of different interacting cell types, endothelial cells (ECs) lining the vessel wall, and perivascular cells or mural cells, mainly composed by pericytes (PC) and vascular smooth muscle cells (vSMC). These processes are crucial during growth and development, injury, repair, and remodeling, since these rely on the interactions between the microvasculature and its microenvironment (Bergers and Song, 2005).

When considering the vasculature that must be regenerated during whole organ tissue engineering, the most crucial and difficult part to engineer is the microvasculature which is composed of ECs and PCs. Moreover larger vessels are surrounded by a layer of vSMCs which regulates the blood afflux to the organ. Determining the best cell source to deliver the different anatomical structures of the vasculature within a whole organ is a crucial step. This section, will focus on the different strategies that have been published in the literature and that have shown potential for use in whole organ tissue engineering.

### Endothelial cells

Finding a suitable endothelial cell source still remains a significant hurdle toward the delivery of vascularized tissue engineered organs. This is particularly significant because adult endothelial cells show an impaired proliferative potential after expansion. In tissue engineering the key aim is to harvest primary cells with minimum invasion, to culture and expand these cells returning a high yield sufficient to colonize the organ. Cells should retain their basic functions, for example endothelial cells should inhibit blood clotting while promoting anastomosis to the host vasculature. With this aim in mind, different cell sources have been investigated ranging from primary isolated adult cells, to cells differentiated from stem or progenitor cell populations.

Classical adult endothelial cell harvesting requires the involvement of large diameter vessels. For this reason, harvesting ECs from patients remains a problem of great concern with significant complications. The only non-invasive sources of adult human ECs remains cadaveric vessels and the umbilical vein (Bourke et al., 1986). Human umbilical vein endothelial cells (HUVECs) have so far been the gold standard in EC research due to their relative ease of accessibility and high yield following isolation. However, HUVECs have shown poor engraftment and anastomosis when transplanted into various animal models. In 2004, with the aim of creating long lasting blood vessels, HUVECs and mesenchymal cells were seeded in a three-dimensional fibronectin-type 1 collagen gel, then implanted into mice. In this experiment, HUVECs were able to form tubes that connected to the host vasculature allowing perfusion. However, HUVECs alone showed limited capacity to form vessels and failed to survive in the long term, although the functionality of the vasculature over a long period of time was not assessed in this study (Koike et al., 2004). More recently Mummery's group observed that HUVECs were not able to incorporate into the vasculature of *zebrafish* xenograft model, but rather attached to the vasculature or migrated throughout the embryo. Therefore, alone HUVECs are an inadequate source of cells for vascular tissue engineering, making it necessary to investigate other options (Orlova et al., 2014).

Endothelial progenitor cells (EPC) can be isolated from the peripheral blood, offering a potential source of autologous cells that can be easily harvested. EPCs were firstly described by Asahara et al. who identified a hematopoietic population capable of eliciting postnatal vasculogenesis in adult peripheral blood (Asahara et al., 1997).

Blood-derived EPCs have already been used to endothelialize synthetic vascular grafts in several studies (He et al., 2003; Shirota et al., 2003). Grafts lined with these EPCs have been implanted *in vivo* into a canine carotid model. After 30 days, 11 out of 12 grafts remained patent, with cells lining the surface showing features of a mature EC phenotype (He et al., 2003). Umbilical cord blood (Murga et al., 2004) and bone marrow (Hamilton et al., 2004) could represent additional sources of autologous vascular progenitor cells. Indeed, several studies have shown that bone marrow–derived cells functionally contribute to neoangiogenesis during wound healing and limb ischemia (Majka et al., 2003), endothelialization of vascular grafts (Shi et al., 1998), and organ vascularization (Otani et al., 2002). Differentiated human umbilical cord blood derived EPCs seeded on vascular scaffolds formed neotissue in both biomimetic and static *in vitro* environments. These tissues were characterized as endothelial monolayers with related functions (e.g., the production of eNOS indicating features of functional endothelium) (Schmidt et al., 2004).

However, significant controversies exist over the identity and role of EPCs in vascular repair, cord blood is not available from all individuals and the rarity and expansion potential of these cells make them unsuitable for scaled-up production. For this reason, alternative sources for patient-specific ECs would be of value.

More recently induced pluripotent stem cells (iPSC) have been broadly investigated as a promising cell source for ECs. Mummery's group found that human iPSC-derived ECs are able to form blood vessels and anastomose to the host vasculature when injected into a zebrafish model (Orlova et al., 2014). Moreover, iPSC-derived ECs were able to outperform HUVECs, which were so far considered the gold standard in EC research. While promising, the clinical use of ECs derived from iPSC is still associated to concerns regarding the tumourogenic potential of pluripotent cells and their limited clinical use for macular degeneration in the retina (Cossu et al., 2017).

It would be advantageous to overcome both the limited yield and availability of adult cells and the tumorougenic potential of EC-iPSC. For this reason, it is possible that the work proposed by Rafii's group would be extremely relevant to the field. In particular, they described the direct conversion of somatic cells to functional endothelium. Interestingly, the approach has been performed with human mid-gestation lineage-committed amniotic fluid derived cells which have been converted into a phenotypically stable and expandable population of vascular ECs without transition through a pluripotent state (Ginsberg et al., 2015).

Recently, the idea that ECs only serve to line simple passive “tubing” systems is slowly coming to an end, their role is more complex than simply delivering oxygen and nutrients, and includes modulating the coagulation of blood, regulating the transportation of inflammatory cells and serving as gatekeepers of cellular metabolism (Carmeliet and Jain, 2011; Ghesquière et al., 2014). Tissue-specific microvascular networks of capillaries can perform complex physiological tasks such as sustaining the homeostasis of resident stem cells and guiding the regeneration and repair of adult organs avoiding fibrosis. Further evidence exists to support the idea that ECs produce angiocrine factor, providing inhibitory and stimulatory tissue-specific signals for stem cell renewal (Butler et al., 2010; Nolan et al., 2013). With this in mind, tissue engineering processes reliant on the use of stem cells could benefit from the establishment of an appropriate endothelial niche. This could provide the ideal environment to recapitulate the complex signaling networks able to instruct organ regeneration.

### Pericytes

ECs are the main component of the vasculature which have been extensively studied and characterized, while pericytes are now coming into focus as key regulators of angiogenesis. Although paternity of pericytes is generally assigned to Rouget in 1874 (Rouget, 1874). Rouget cells have since then been re-named pericytes, referring to their anatomical localization in close proximity to the endothelial layer by Zimmermann in 1923 (Zimmermann, 1923). The periendothelial location of pericytes is frequently confused with the periendothelial location of vascular smooth muscle cells, fibroblast and macrophages (Armulik et al., 2011). Although everyone adopted the view that pericytes belong to the same lineage of vSMCs, it is widely accepted that there is no singular molecular marker that enables us to distinguish them unequivocally from vSMCs or other mesenchymal cells. In addition, the expression of markers used to identify pericytes is transient and is not consistent, indeed different perycites can express different sets of markers and this expression can change throughout the life of the same cell (Armulik et al., 2011). Due to this heterogeneity, and marker promiscuity, it has been impossible to fully establish pericyte identity, and the only clear definition refers to their anatomical location. Currently cells defined as pericytes are localized in the vascular basal membrane as seen via electron microscopy (Miller and Sims, 1986).

However, this definition loses strength in conditions of active angiogenesis, such as during embryogenesis and tissue regeneration, where clear identification of these pericytes becomes even more difficult. It is also widely accepted that pericytes are more frequent in the proximity of micro-vessels (capillaries, venules, and terminal arterioles), where they share the basal membrane with endothelial cells, and are connected by tight, gap, and adherent junctions. Indeed, a single pericyte can be connected with several endothelial cells by cell protrusions that wrap around, and along the blood vessel (Gerhardt and Betsholtz, 2003; Kovacic and Boehm, 2009). However, even this definition has been challenged by the observations of sub-endothelial pericyte-like cells in large vessels (Díaz-Flores et al., 2009). Although many controversial aspects exist in the field, an increasing number of studies suggest that perycites may be the progenitor of vSMCs, and may constitute multipotent progenitor cells like adipocyte progenitors (Olson and Soriano, 2011), osteoblast, chondrocytes (Collett and Canfield, 2005), and skeletal muscle stem cells (Dellavalle et al., 2007). This resembles the behavior of mesenchymal stem cells (MSCs) and therefore it has led to the concept of a perivascular niche of MSCs (Armulik et al., 2011).

Paolo Bianco's laboratory has argued in support of this theory, and on the widely shared view that MSCs are ubiquitous in human connective tissue, defined by common *in vitro* phenotype and coinciding with ubiquitous pericytes. They reported that ubiquitous MSCs with identical capacities do not exist, but that “tissue-specific” mesodermal progenitors are capable of being recruited to a mural cell fate, providing a plausible mechanism by which pericytes are formed, and how they serve as a source of local progenitor cells (Sacchetti et al., 2016). Together these considerations provide evidence to suggest that pericytes are a powerful tool for tissue regeneration since they can contribute to restoration of the vascular smooth muscle layer, known to be essential for a functional and mature endothelium (Bergers and Song, 2005), as well as the mesodermal compound of the tissue from which they originate. Nevertheless, it is not possible to exclude the theory that these cells can contribute to regeneration of the smooth muscle compound of organs due to their default capacity to differentiate into vSMCs, which does not have a clear distinction from non-vascular smooth muscle cells.

Regardless their origin, it is largely accepted that perivascular cells play an important role during the early phases of angiogenesis. Although initial endothelial cell sprouts may form without pericyte involvement, pericytes are among the first cells to invade newly vascularized tissues, and are found to be located at the growing front of endothelial sprouts. Pericytes can suppress endothelial growth, migration and microvessel stabilization (Bergers and Song, 2005; von Tell et al., 2006), moreover, pericyte involvement has also been directly implicated in conferring capillary resistance to regression *in-vivo*. For this reason, an increasing number of studies are focusing on these cells type for the purpose of vascular tissue engineering. As a source of pericytes for tissue engineering, a pioneering study from the groups of Bianco and Cossu reported that cells isolated from the embryonic murine dorsal aorta, and ascribed to the perivascular lineage (by the expression of CD34, Flk-1, SMA and c-Kit), are able to generate *in-vivo* both vascular and extravascular mesodermal derivatives. For this reason, these cells have been named mesoangioblasts (MAB) (Minasi et al., 2002). However, MABs derived from adult tissue lose their endothelial features, and are therefore considered “pericyte derived” cells. Cells with similar features can be isolated from skeletal muscle biopsies, and they are able to differentiate down the smooth muscle lineage (default function of a pericyte) and skeletal muscle (as mesodermal lineage of origin) (Dellavalle et al., 2011). Similarly to what we have discussed above, pericytes could be derived from pluripotent stem cells. A protocol for deriving MAB-like stem/progenitor cells from human and murine iPSCs has been recently established (Gerli et al., 2014). Relevantly, defined conditions for simultaneous derivation of ECs and PCs from hiPSCs of different tissue origin with high efficiency have also been defined (Orlova et al., 2014).

### Vascular smooth muscle cells

From a whole organ tissue engineering perspective, the overall range of differently sized vessels which form the vascular tree must be regenerated because microvasculature alone cannot support organ function. Indeed, smooth muscle cells, which represent the main difference between microvasculature and larger vessels, have a crucial role in delivering vasculature function. They deliver vasomotiliy and contribute to the biomechanical blood flow response (Neff et al., 2011). Consequently, derivation and culture of vascular smooth muscle cells represent a significant step toward regeneration of the whole vasculature.

Different approaches have been exploited in order to find a suitable and reliable cell source that could give rise to the vascular smooth muscle compartment. However, while in the specific field of blood vessels tissue engineering, intended to regenerate a single vascular graft, a lot of efforts have been put into engineering the smooth muscle layer (Tresoldi et al., 2015), the field of whole organ revascularization has mainly addressed pericytes regeneration to provide a stable microvasculature.

Mesenchymal stem or stromal cells are one of the most widely investigated sources to derive VSMCs. A seminal paper which combined decellularized scaffold and MSCs was published by Zhao et al. They were able to derive ECs and vSMCs from bovine MSCs to fully develop tissue engineered arteries which were transplanted into a sheep model as a carotid artery interposition which remained patent over 5 months (Zhao et al., 2010a). Another example is provided in the work by Jung et al. where human MSCs were used to create a scaffold-free graft which featured a mature smooth muscle layer (Jung et al., 2015).

Beside MSC, adipose derived stem cells (ADSCs) represent another broadly exploited VSMCs source because of their easy harvesting. Indeed this cell type is able, under the right biochemical and biomechanical conditions, to give rise to a mature smooth muscle phenotype that features contractility (Harris et al., 2011). The principal pathway involved seems to be transforming growth factor-beta 1 (TGF-β1). Indeed, in a similar study, ADSCs were induced to differentiate into VSMCs through TGF-β1 and bone morphogenic growth factor. The human VSMCs derived from ADSCs were seeded on small-caliber vascular graft. The resulting vessel wall had a dense and well-organized structure similar to the one of physiological vessels (Wang et al., 2010).

A remarkable example of delivering the smooth muscle layer in a whole organ is represented by the promising results that have been achieved by Ott's laboratory. In their work, the co-seeding of HUVECs and human MSCs, as perivascular supporting cells, in decellularized rat lung scaffolds resulted in a broad re-endotheliasation, but more interestingly, in the same work, they regenerated the lung vasculature using both endothelium and vSMCs with cells derived from human inducible pluripotent stem cells, showing how these cells can represent another valuable source of vSMCs (Ren et al., 2015).

Furthermore, vSMCs, as mentioned above, are supposed to feature a common ancestry with pericytes, making the sources of PCs presented in the previous paragraph a potential cell population for the derivation of the smooth muscle phenotype. In particular, mesoangioblasts, which are an easily accessible cell population are already used in the clinic (Cossu et al., 2015), and can give rise to a smooth muscle phenotype (Tagliafico et al., 2004), making them an ideal candidate for regeneration of the smooth muscle vascular layer. Another option which has been investigated, is the direct recruitment of VSMC *in-vivo*. This approach worked for blood vessels of 5–6 mm in diameter (Pellegata et al., 2015; Syedain et al., 2017).

To summarize, a fully functional and mature vasculature requires both endothelial and mural cells. Many studies focused on blood vessel tissue engineering have demonstrated the importance of this coexistence. However, the majority of the attempts to regenerate the vasculature in whole organs have been carried out using only ECs as discussed in the next sections of this review. The results achieved in the last years, using different cell types together, definitely suggest that the co-culture approach is more appropriate to address whole organ revascularization.

## Liver and pancreas

Within the field of whole organ tissue engineering, liver is the most widely investigated organ of all, with one third of studies published on the liver including the issue of vascularization. Indeed, there is a relevant clinical need dictated by the shortage of available organs for transplant. Moreover, the anatomy of the liver lends itself well to decellularization, a process that has been established for both animal and human liver (Mazza et al., 2015). Subsequent recellularization can be obtained through the portal vein, even in small animal models. Indeed, the portal vein together with suprahepatic vein represents the most relevant vasculature since a liver can survive without its arterial supply. Hepatic tissue engineering still remains a significant challenge and to date the derivation of mature hepatocytes is an unmet goal (Hannan et al., 2013; Leclerc et al., 2017), however the environment, in particular the decellularized matrix seems to favor this process (Lorvellec et al., 2017). Potentially endothelial cells could play an active part in liver tissue engineering, since they are known to control liver regeneration (Ding et al., 2010), indeed Taniguchi's group described in a pioneering study that the transplantation of vascularized liver buds were able to recapitulate the organ function (Takebe et al., 2013).

From an organ tissue engineering perspective, the main strategy adopted is the injection of endothelial cells, mainly HUVECs (Baptista et al., 2011; Shirakigawa et al., 2013; Takebe et al., 2014; Bao et al., 2015; Verstegen et al., 2017). Pioneering work was performed by Soker's group in 2011 where they reported decellularization of whole livers from different species. In addition, the group cultured HUVECs together with fetal cells in ferret liver scaffolds and demonstrated that the regenerated endothelium does not leak when perfused with labeled dextran (Baptista et al., 2011). Bao et al. cultured HUVECs in pig decellularized scaffolds functionalized with heparin over 3 days, showing a good engraftment of the cells, furthermore they showed how the functionalized decellularized liver does not elicit sudden thrombosis when grafted in the infra-hepatic space of piglets (Bao et al., 2015). Orthotopic liver transplant has been performed also by Atala's group, in their study MS1 re-endothelialized porcine livers sustained blood perfusion *in-vivo* for 24 h avoiding thrombi formation and resulting in patent vessels as confirmed by ultrasound monitoring (Ko et al., 2015). HUVECs were used also in another study, in which endothelial cells were dynamically cultured for 3 days on decellularized rat livers, interestingly the cells prevented blood leakage upon *in-vitro* reperfusion (Shirakigawa et al., 2013). HUVECs were also tested on human decellularized livers, specifically Verstegen et al. seeded HUVECs on 250 μm scaffold slices achieving a good recellularization and highlighting how the matrix instructed cells to locate in the vascular tissue (Verstegen et al., 2017). Furthermore, in another study the endothelial cell line EA HY926 was used to recellularize an acellular pig liver lobe, coated with heparin gel which promoted cell adhesion and engraftment. Dynamic culture for 10 days resulted in an even cell distribution of cells, and the scaffold did not elicit thrombosis when heterotopically grafted in pigs for 1 h (Hussein et al., 2016). It should be remarked that in the studies by Hussein et al. and Bao et al. the addition of heparin immobilized on the scaffold enhanced the blood perfusion over time *in-vivo* (Bao et al., 2015; Hussein et al., 2016). Perfusion over the long term still remains the main hurdle, indeed these studies showed a limited time frame of up to 3 days post-implantation, while patency over a long period should be established to deliver a clinically relevant hepatic graft. Kadota et al. proposed an approach in which bone marrow derived mesenchymal stem cells seeded in a rat liver produced pro-angiogenic factors and sustained an orthotopic transplantation for 60 min (Kadota et al., 2014). This represents a reversed approach in which the strategy is to promote the ingrowth of host vasculature, similar to what is described in similar studies using collagen gel, and showing ingrowth of host blood vessels. In particular, in the study by Zhao et al. the collagen gel was embedded with hepatocytes before subcutaneous implantation for 7 days (Takimoto et al., 2003; Zhao et al., 2010b). However, this approach limits the size of the graft as the ingrowth of blood vessels cannot be fast enough to sustain human sized tissues while avoiding necrosis.

The pancreas, which shares many key endocrine and exocrine functions with the liver, has been decellularized as a whole organ to address the possibility of islet transplantation. Two reports have been published which have addressed recellularization with endothelial cells. Peloso et al. reported the dynamic culture of human decellularized pancreas with human primary pancreatic endothelial cells (Peloso et al., 2016). Results showed engraftment of cells which maintained a CD31 positive phenotype, half of the cells seeded continued to proliferate. Furthermore, Guo et al. recently performed re-endothelialization of decellularized rat pancreas with EPCs (Guo et al., 2018). The repopulated scaffolds were subcutaneously implanted in mice showing anastomosis with host vasculature resulting in a higher density of blood vessels in the graft compared to unseeded scaffolds.

To go progress liver and pancreas tissue engineering for translation into clinical care, it will be necessary to deliver pre-vascularized engineered organs which could sustain long-term orthotopical engraftment. Focus should be given to engineer specialized liver endothelium which features a very particular and site specific vasculature that should be recapitulated.

## Kidney

The kidney contains clear vascular access from both arterial and venous vessels. As a filtration organ, vasculature plays a key role in organ function, making the vasculature necessary not only for the survival of the engineered graft, but also to deliver its function. Different cellular sources have been used to engineer the renal vasculature, with the renal artery being the main route of cell delivery.

The first attempt at whole kidney tissue engineering was described by Ott's group. In their study they described the decellularization of kidneys from different species, such as rat, pig and human. They were able to repopulate rat kidneys using HUVECs and rat neonatal kidney cells, achieving a spatial distribution that resembled the native glomerula. Dynamic culture of the seeded cells resulted in a drop in vascular resistance and evidence of vascular function. The group performed a short-term orthotopic implantation of the engineered kidney by anastomosis of both vascular pedicles. They reported evidence of a distinct vasculature which avoided the formation of blood clots, although it was not clearly stated for how long the graft was in place (Song et al., 2013). Interestingly, they reported that the extracellular matrix was able to spatially instruct the cells and drive them toward the correct anatomical location.

The notion of an instructive ECM has been also described by Remuzzi's group. In their work they showed that murine embryonic stem cell fate is influenced by the decellularized scaffold. In this experiment, cells seeded through the renal artery of a decellularized rat kidney became distributed evenly in capillary structures. Within 72 h of seeding the cells had lost their pluripotency, and were shown to differentiate into mesoderm-derived endothelial precursors (Bonandrini et al., 2014). The instruction of embryonic stem cells has also been demonstrated in large animal studies. In a study proposed by Batchelder and colleagues, human ESCs were seeded on decellularized rhesus monkey kidneys. After 7 days, the cells differentiated into kidney specific cell types comprehensive of a CD31+ cell fraction (Batchelder et al., 2015). To further describe how endothelial cells could orchestrate cell fate, Du et al. seeded a mouse decellularized kidney with iPSC-derived Pax-2+ progenitors and iPSC-derived endothelial cells. They showed how the presence of endothelial cells regulated the expression of renal genes in the progenitor cells. The scaffolds were subcutaneously implanted in SCID mice for 12 weeks. Results showed that only in the presence of endothelial cells were the glomeruli recellularized. Moreover, they showed *in-vitro* how endothelial cells improved glomerular barrier function (Du et al., 2016). Very recently Bombelli et al. derived human nephrospheres, the spheres were shown to contain renal stem cell like cells. Interestingly, when cultured on decellularized tissue slices they showed that the ECM is again able to instruct the nephrospheres and drive their differentiation toward endothelial cells and tubular structures in 30 days (Bombelli et al., 2017). Overall kidney revascularization has been investigated using a wide range of different cell sources, demonstrating that the extracellular matrix can provide the cues required for cell differentiation. Alternatively, Atala's group has focused on primary cells, providing evidence which suggest that primary cells can be used to replace human size renal function and they have developed a method that allows the efficient expansion of primary cells which can maintain a normal renal phenotype (Abolbashari et al., 2016).

## Intestine

Decellularization has proven to be an effective technique to provide acellular intestinal scaffolds, with preserved native vasculature (Totonelli et al., 2012). Preserving vascular access such as the mesenteric artery and the mesenteric vein facilitates transplantation of the organ, providing vessels that can be anastomosed in the host, providing immediate perfusion to the organ, essential for survival of the graft upon transplantation (Zhu et al., 2008). To date, a very limited number of papers have addressed the vasculature of the intestine. Very recently, decellularized rat intestinal segments were engineered with iPSC-derived epithelial cells for 14 days and HUVECs for 3 days. The intestine was subsequently implanted in a heterotopic model consisting of a subcutaneous graft in the neck, anastomosed to the vasculature and provided with two-end stomas. The graft was maintained for 4 months, results reported graft functionality, and nutrients delivered into the lumen of the engineered intestine, via the stoma, were adsorbed into the rat blood stream. However, patency of the engineered blood vessels wasn't directly assessed (Kitano et al., 2017). A similar approach was taken by MacNeil's group, who injected human dermal microvascular endothelial cells, and human dermal fibroblasts, into the vasculature of decellularized rat intestines. They demonstrated successful delivery and engraftment of the cells in the decellularized vasculature, in addition to ongoing sprouting angiogenesis featuring DLL4 positive cells (Dew et al., 2016). To date there have been few reports investigating the topic of intestine re-endotehlialization. Intestinal tissue engineering is being studies by many groups but the development of intestinal vasculature still remains an open field that deserves attention. The intestinal epithelium is a complex environment in which there exists a complex crosstalk between many different cell types, it will thus be fundamental to unveil how epithelium and endothelium interact in orchestrating this particular epithelium. Finally, it is relevant to remark that in order to achieve a functional intestine, engineering of the lymphatic tissue is essential and this has only be partially explored (Koike et al., 2004).

## Lung

Lung function depends on the presence of a functional vasculature to achieve optimal gas exchange. Therefore it is not surprising that nearly half of the work related to whole lung engineering is focussed on engineering of the vasculature. Early studies demonstrated the feasibility of whole lung decellularization (Maghsoudlou et al., 2013). Cortiella et al. reported the first attempt to decellularize a whole lung. In this study, they showed how the pulmonary extracellular matrix was able to instruct mouse ESCs to differentiate into site-specific cells such as CD31+ cells, similarly to what has been seen in ECMs derived from other organs (Cortiella et al., 2010). The same group recently reported the decellularization of human pediatric lungs, with subsequent seeding of primary adult human epithelial and vascular cells, and dynamic bioreactor culture (Nichols et al., 2017). Interestingly, they reported a good distribution of cells throughout the scaffold, with proper recellularization of the blood vessel. Type 1 and 2 epithelium were present, and were shown to have the capacity to produce surfactant.

In 2010, Niklason's team decellularized rat lungs and seeded them with pulmonary epithelium and vascular endothelium utilizing a custom-made bioreactor. Interestingly, the seeded epithelium displayed remarkable hierarchical organization within the matrix, and the seeded endothelial cells efficiently repopulated the vascular compartment. Moreover, mechanical characteristics of the engineered lungs were similar to those of native lung tissue, and when implanted into rats the engineered lungs participated in gas exchange (Petersen et al., 2010). Ott's group has a long history of work addressing whole lung engineering; in 2014 they reported decellularization of rat, pig and human lungs, and the cytocompatibility of these matrices by seeding either epithelial cells or HUVECs on slices (Gilpin et al., 2014). In a subsequent study, they introduced the concept of addressing both the endothelial and the perivascular compartments, and introduced seeding through both arterial and venous routes (Ren et al., 2015). They co-seeded either HUVECs and hMSC, or iPSC-derived ECs and PCs, reaching a 75% endothelial coverage that resulted in a good barrier function. Re-endothelialized lungs were orthotopically transplanted into rats for 3 days, *in-vivo* characterization was limited to showing the presence of cells and perfusability of the HUVECs-hMSC seeded grafts. Finally, in the same study they showed scalability, up to human lung size. Scalability was improved in two later studies in which the same group showed the recellularization of human and pig lungs with both epithelial and endothelial cells (Gilpin et al., 2016; Zhou et al., 2017). However, the latter were orthotopically transplanted for only 1 h showing a poor degree of gas exchange. Other published studies have been able to reach an even cellular distribution throughout whole decellularized rat lungs, following seeding using rat microvascular endothelial cells (Calle et al., 2016; Stabler et al., 2016). Niklason's group exploited the concept of regenerating the vessel mural compartment by re-endothelializing decellularized rat lungs using rat endothelial cells supported by rat adipose-derived stem/stromal cells which gave rise to pericytes. The re-endothelialized lungs showed improved vascular resistance comparable to native lungs, moreover they were orthotopically transplanted in rats for 3 h showing how the presence of perivascular cells avoided oedema formation (Doi et al., 2017).

Finally, a slightly different approach was proposed by Wagner et al. (2014). In their study, they isolated bronchovascular bundles and coated these with sodium alginate showing how this increases cellular adhesion toward scaling up, however, they report variability in the decellularization outcomes that failed to be standardized. Taken together studies focused on the lung show how seeding through both arterial and vascular vessels is necessary in order to reach an even distribution of cells, that can potentially support organ function. Results are encouraging, however, lung re-endothelialization still lacks *in-vivo* transplantation results which exceed 3 days.

## Heart

In the context of the heart, endothelial cells play a double role, both lining the coronary vasculature, providing nutrients to the hearth muscle, as well as covering the valves and chambers of the heart. Whole heart re-endothelialization has been firstly investigated in the rat by Taylor's team. Rat aortic endothelial cells were seeded onto decellularized rat hearts via perfusion of the aorta. After 1 week of dynamic culture in a bioreactor, cells repopulated coronaries and showed metabolic activity (Ott et al., 2008). A similar approach was described by Yasui et al. who reported 30 days of successful dynamic co-culture when scaffolds were seeded with rat neonatal endothelial cells alongside rat neonatal cardiomyocytes and fibroblasts. These results showed that, although they achieved contraction, cells were randomly distributed in the organ ECM (Yasui et al., 2014). From a methodological perspective, other studies have compared different seeding approaches. Robertson et al. demonstrated that when rat aortic endothelial cells are seeded onto a decellularized rat heart, better cell distribution is achieved when perfusion is via the inferior vena cava and brachiocephalic artery compared to the aorta. After 7 days, of bioreactor culture cells are shown to retain their phenotype, were able to produce nitric oxide, and reduced thrombosis in an *in-vitro* assay. Finally, they performed a heterotopic transplantation by anastomosis to the aorta and inferior vena cava of the recipient, transplanting unseeded scaffolds as control. After 1 week, re-endothelialized hearts showed clear vessel formation with reduced incidence of blood clotting (Robertson et al., 2014).

Scaling up of techniques has been described in two studies using pig and human hearts. Weymann et al. seeded HUVECs and murine neonatal cardiac cells onto the decellularized scaffold of a pig heart, and cultured the organ in a bioreactor for 3 weeks. After 10 days a homogeneous re-endothelialization of the coronary tract was reported (Weymann et al., 2014). Sanchez et al. reported the decellularization of a whole human heart (Sánchez et al., 2015) followed by recellularization of tissue slices. Recellularization was tested by culturing HUVECs with different cell types on scaffold slices for 21 days. Endothelial cell migration was reported and cells were shown to line the endocardium and vasculature, demonstrating again that the ECM can direct cells to their correct location during engraftment. To successfully engineer a whole heart for clinical translation, scaling up of techniques is essential. Studies to date are limited to the use of HUVECs and rat aortic cells, making it necessary to find a more suitable primary cell source, especially because endothelium is needed also for atria, ventriculi, and valves. Heart orthotopic engineering poses incredible challenges, heterotypical implants in addition to proper functional evaluation of the *in-vitro* engineered vasculature are required to proceed to the next step.

## Discussion and future directions

To date, there is significant interest in the field of whole organ tissue engineering. The growing demand for a solution to the availability of transplant organs is high on the agenda for patients, clinicians and health care providers. The field of whole organ engineering is an innovative and exciting area of research with the potential to overcome, and provide a solution to the availability of transplantable organs. There are some organs and tissues for which the vasculature does not play a prominent role, as demonstrated by successful transplantation of engineered tracheas which were supported by an omental wrap at orthotopic transplantation (Elliott et al., 2012). As discussed above however, it is clear that the vasculature is a fundamental feature of more complex organs.

The majority of studies on-going in the field of whole organ tissue engineering take advantage of decellularized organs as the basis, providing a scaffold on which the complex multicellular organ can be built. This is not surprising as, to date, no other manufacturing method can deliver a scaffold that can recapitulate the complex structure and anatomy of a human organ. It can be speculated that in the near future innovations in manufacturing techniques, such as 3D printing or stereolithography, will be able to provide complex structures using bioactive materials. Indeed, the advances in 3D bioprinting may directly benefit the field of tissue engineering by allowing the direct printing of cells onto a scaffold.

Nevertheless, current decellularization techniques are able to preserve the architecture of whole organs, particularly the vasculature, resulting in the possibility of delivering the endothelial cells directly through perfusion, allowing for the creation of a vascular network that is similar to the primary organ.

In contrast to a synthetic scaffold, naturally derived scaffolds maintain the extracellular matrix which has been shown to have a positive influence on cell seeding, promoting cell engraftment, migration and differentiation. This feature of the ECM has been demonstrated by different groups internationally, and represents one of the most powerful tools in the hands of researchers. Currently, we do not fully understand the biochemical and topological cues that drive this process, however cells can be organized and moved to the right location even in complex and highly tissue-specific sites. From a methodological perspective, exploiting this feature of the extracellular matrix researchers can deliver different cell populations to a decellularized scaffold simultaneously, relying on self-arrangement.

The main hurdle to tissue engineering complex multicellular organs is the culture environment. Different cell types require different biochemical environments and stimuli, identifying a culture medium that is well tolerated by a variety of cells of different origins, without influencing differentiation pathways poses a significant challenge.

Beside biochemical and topological cues, a dynamic environment plays a crucial role in tissue engineering, this is demonstrated by the benefit provided by bioreactor based dynamic culture. This is particularly important for the vasculature and it is mandatory to provide perfusion to engineer blood vessels. Endothelial cells strongly benefit from shear stress, to grow and express the correct phenotype. Calculating the amount of shear stress provided in an intricate blood vessel network such as that of a whole organs is complex. The beneficial effect of perfusion is clear, at least in term of providing an even distribution of nutrients. Complex computational models, coupled with particle tracking, could be applied to this field to precisely tune the amount of shear stress the endothelial cells need to be exposed to. Moreover, providing a dynamic perfusion avoids the formation of cell clumps during culture.

Taken together the studies reviewed here hold great potential that require further exploitation and scale up. However, there is still a significant gap toward real clinical translation of engineered revascularized organs. Many studies to date, only exploit one vascular access route and do not investigate whether both venous and arterial networks are recellularized. The few groups who have exploited the infusion of cells through both arterial and vascular accesses showed a more homogeneous distribution of the cells throughout the scaffold and to the authors this looks like the path to follow in future seeding strategies. Besides cell seeding, no studies have demonstrated the ability of long-term perfusion in orthotopic models, current studies report short time points limited to a few hours or days, mainly because of blood clotting. Furthermore, studies should focus on providing reliable and reproducible methods to assess vasculature function and patency. Active methods that show perfusion of the organ should be used and patency assessed *in-vivo*. To date no studies have achieved a fully confluent regenerated endothelium, and this remains a significant hurdle in demonstrating functionality resembling the normal physiological state.

In order to reach long term patency of the blood vessel network, researchers should focus on delivering *ex-vivo*, a stable and functional vasculature that provides an adequate and confluent endothelial layer. Research has been hampered by the availability of clinically relevant endothelial cell sources. Almost the majority of the studies presented in this review use HUVECs, but adult endothelial cells have a limited proliferative potential and are not stable over long time periods (Table 1). This largely limits the application of normal adult cells for human sized tissue. iPSCs can be a powerful alternative, but limited by the current concerns on safety and the difficultly in achieving a functional, stable, and homogenous differentiation. Conversely, the direct reprograming of adult cells, taking advantage of the expression of fetal factors, holds a great potential with less procedural concerns. As it has been highlighted, perivascular cells exert a fundamental role in the development and stabilization of vasculature. Considering that reliable and non-invasive sources of perivascular cells, such as mesoangioblasts are available, tissue engineering studies should put efforts into developing strategies that take advantage of using perivascular cells to support endothelial cells.

**Table 1:** Summary of the studies presented in the review addressing revascularization in whole organs.

**Organ**	**Cells**	**Scaffold**	**Seeding method**	***In-vitro* culture**	***In-vivo***	**References**
Liver	EA HY926 endothelial cell line and HepG2	Decellularized pig lobe	Perfusion in portal vein and hepatic artery	Dynamic 10d	Heterotopic 1 h	Hussein et al., 2016
Liver	HUVECs	Decellularized pig scaffold	Perfusion in portal vein	Static 3d	Orthotopic in infrahepatic space for 1 h	Bao et al., 2015
Liver	Endothelial progenitor cells	Decellularized rat liver	Perfusion in portal vein	Dynamic 3d	Subcutaneous 21d	Zhou et al., 2017
Liver	HUVECs and human fetal liver cells	Decellularized mouse, rat, ferret, rabbit and piglet livers	Perfusion in portal vein an vena cava	Dyanmic 7d	Scaffold reperfusion in terminal anesthesia	Baptista et al., 2011
Liver	MS1	Decellularized pig liver	Perfusion in portal vein	Dynamic 3d	Orthotopic 24 h	Ko et al., 2015
Liver	HUVECs	Decellularized human liver slices	Top seeding	Static 5d	–	Verstegen et al., 2017
Liver	Bone marrow MSC and hepatocytes	Decellularized rat liver	Perfusion in portal vein	Dynamic 6d	Orthotopic 60 min	Kadota et al., 2014
Liver	HUVECs and hepatocytes	Decellularized rat liver right lobe	Perfusion in portal vein	Dynamic 3d	–	Shirakigawa et al., 2013
Pancreas	Human primary pancreatic endothelial cells	Decellularized human pancreas	Perfusion in superior mesenteric artery and splenic artery	Dynamic 6d	–	Peloso et al., 2016
Pancreas	Rat EPC	Decellularized rat pancreas	Perfusion in inferior vena cava	Dynamic 3d	Subcutaneous in mice 20d	Guo et al., 2018
Kidney	Mouse ESC	Decellularized rat kidney	Perfusion in renal artery	Dynamic 72 h	–	Bonandrini et al., 2014
Kidney	HUVECs and rat neonatal kidney cells	Decellularized rat, pig and human kidneys	Vacuum assisted perfusion in renal artery	Dynamic 5d	Orthotopic in rats	Song et al., 2013
Kidney	iPSC derived Pax-2 progenitors and endothelial cells	Decellularized mouse kidney	Perfusion in renal artery	Static 16 h	Subcutaneous in SCID mice for 12 weeks	Du et al., 2016
Kidney	Human neurospheres	Decellularized human kidney slices	Top seeding	Static 30d	–	Bombelli et al., 2017
Kidney	Human ESC	Decellularized rhesus monkey kidney	Perfusion in renal artery	Dynamic 7d	–	Batchelder et al., 2015
Lung	Human lung epithelial and pulmonary endothelial cells	Decellularized rat and human lung	Perfusion in pulmonary artery	Dynamic 7d	–	Gilpin et al., 2016
Lung	HUVECs and human epithelial cells	Decellularized rat, pig and human lung slices	Top seeding	Static 5d	–	Gilpin et al., 2014
Lung	HUVECs and hMSC or iPSC derived endothelial cells and perivascular cells	Decellularized rat lung and human lung single lobe	Perfusion in pulmonary artery and vein	Dynamic 6d	Orhotopic in rats for 3 days	Ren et al., 2015
Lung	HUVECs and human airway epithelial progenitors	Decellularized pig lung	Perfusion in pulmonary artery and vein	Dynamic 6d	Orhotopic in pigs for 1 h	Zhou et al., 2017
Lung	Mixed rat neonatal lung population and rat lung microvascular endothelial cells	Decellularized rat lung	Perfusion in pulmonary artery and vein	Dynamic 4d	–	Calle et al., 2016
Lung	Rat microvascular endothelial cells	Decellularized rat lung	Perfusion in pulmonary artery	Dynamic 7d	–	Stabler et al., 2016
Lung	Human mixed lung population	Decellularized human pediatric lung pieces	Top seeding	Dynamic 7d	–	Nichols et al., 2017
Lung	Rat ECs and ADSC	Decellularized rat lung	Perfusion in pulmonary artery and vein	Dynamic 16d	Orhotopic in rats for 3 h	Doi et al., 2017
Lung	Human adult cells	Bronchovascular bundles isolated from decellularized pig lung and human lobe	Perfusion in the bundle	Static 28d	–	Wagner et al., 2014
Lung	Mouse ESC	Decellularized rat lung	Perfusion in the bronchi	Dynamic 21d	–	Cortiella et al., 2010
Heart	Rat aortic endothelial cells and rat neonatal cardiac cells	Decellularized rat heart	Injection	Dynamic 7d	–	Ott et al., 2008
Heart	Rat neonatal endothelial cells, cardiomyocytes and fibroblasts	Decellularized rat heart	Perfusion in the aorta	Dynamic 30d	–	Yasui et al., 2014
Heart	Rat aortic endothelial cells	Decellularized rat heart	Retrograde aortic, brachiocephalic artery or IVC and brachiocephalic artery perfusion	Dynamic 7d	Heterotopic 7d	Robertson et al., 2014
Heart	HUVECs, human cardiac progenitors, hMSC or cardiomyocytes	Decellularized human heart slices	Top seeding	Static 21d	–	Sánchez et al., 2015
Heart	HUVECs and murine neonatal cardiac cells	Decellularized pig heart	Perfusion in the aorta	Dynamic 21d	–	Weymann et al., 2014

In conclusion, the fields of whole organ tissue engineering has reached the time of scaling up to develop functional human sized preclinical models. Vascularization will be the cornerstone essential for the generation of fully functional, tissue engineered organs which will survive and function post-transplantation.

## Author contributions

AFP, AMT, and PDC contributed with conception and writing of the paper.

### Conflict of interest statement

The authors declare that the research was conducted in the absence of any commercial or financial relationships that could be construed as a potential conflict of interest.
